# Case Report: Expanding the diagnostic spectrum of non-invasive prenatal testing to structural chromosomal abnormalities

**DOI:** 10.3389/fgene.2026.1746287

**Published:** 2026-02-17

**Authors:** Jong Chul Kim, Hyunjin Kim, HeeYeon Jang, Minyeon Go, Ji Eun Park, Chang Soo Ryu, Mi Uk Chin, Eun Hye Kim, Young Jin Lee, Sung Han Shim, Dong Hyun Cha

**Affiliations:** 1 Center for Genome Diagnostics, CHA Biotech Inc., Seoul, Republic of Korea; 2 Department of Biomedical Science, College of Life Science, CHA University, Seongnam-si, Republic of Korea; 3 Department of Obstetrics and Gynecology, CHA Gangnam Medical Center, CHA University, Seoul, Republic of Korea

**Keywords:** chromosomal microarray, karyotyping, non-invasive prenatal testing, sex chromosomal aneuploidies, structuralchromosomal abnormalities

## Abstract

Non-invasive prenatal testing (NIPT) has recently expanded to include sex chromosomal aneuploidies (SCAs) and copy number variations (CNVs), as well as the commonly screened trisomies (T21, T18, and T13). While the clinical utility of NIPT for detecting common fetal chromosomal aneuploidies is well established, its application in assessing structural chromosomal abnormalities (StrCAs) remains controversial, with limited consensus within the medical community. Furthermore, the accuracy of NIPT for detecting SCAs and CNVs is relatively lower than that for common trisomies. This study reports three cases in which NIPT results suggestive of SCAs were clarified by invasive diagnostic testing to represent underlying structural sex chromosome abnormalities. NIPT results suggestive of SCAs were validated through invasive diagnostic tests, including karyotyping, chromosomal microarray (CMA), quantitative fluorescence PCR (QF-PCR), and multiplex ligation-dependent probe amplification (MLPA). In the first case, the NIPT result suggestive of a monosomy X-like pattern reflected an underlying structural abnormality. Fetal chromosomal microarray (CMA) revealed a 3.6 Mb deletion involving the Xq27.3–q28 region and a 4.8 Mb duplication encompassing Xq28, with subsequent analysis confirming inactivation of the deleted X chromosome. In the second case, the NIPT result suggesting monosomy X with a low Y chromosome fraction (1.46%) resembled a vanishing twin pattern but was ultimately explained by mosaicism involving a ring Y chromosome (46,X,r(Y)/45,X). CMA revealed a 7.4 Mb duplication of Yp11.31–p11.2 and a 15 Mb deletion of Yq11.21–q11.23, confirming mosaic ring Y formation. In the third case, the NIPT finding suggestive of XYY with Xp22.33–p22.2 deletion was clarified by confirmatory testing as a maternal sex chromosome translocation, 46,X,der(X)t (X; Y) (p22.2; q11.222), detected in the mother with short stature but no other clinical features. In conclusion, these three NIPT findings initially interpreted as SCAs were clarified by confirmatory invasive diagnostics, illustrating the complexity of interpreting results associated with StrCAs. These findings support the potential of NIPT to extend beyond numerical aneuploidy screening and contribute to the detection of structural chromosomal abnormalities.

## 1 Introduction

Sex chromosome abnormalities refer to numerical or structural abnormalities of the sex chromosomes, including full or partial duplications or deletions. Common sex chromosome aneuploidies (SCAs) include 45,X, 47,XXX, 47,XXY, and 47,XYY, while rarer forms such as 48,XXXX and 49,XXXXX occur less frequently. The discovery of cell-free DNA (cfDNA) by Lo et al. and advances in next-generation sequencing (NGS) led to the introduction of massively parallel sequencing-based noninvasive prenatal testing (NIPT) by Palomaki et al., initially targeting common trisomies and SCAs (Lo et al., 1997; Palomaki et al., 2011). cfDNA in maternal plasma contains not only maternal cfDNA but also cfDNA derived from the placental trophoblast, allowing for the identification of fetal chromosomal abnormalities. The cfDNA-based screening approach significantly reduces the need for invasive testing and mitigates the associated risks of maternal anxiety and miscarriage. NIPT is now widely adopted due to its high sensitivity and specificity for detecting common aneuploidies. In a large cohort study by Zhang et al., the sensitivity of NIPT for trisomy 21, 18, and 13 was reported as 99.17%, 98.24%, and 100%, respectively, with corresponding specificities of 99.95%, 99.95%, and 99.96% (Zhang et al., 2015). In contrast, the positive predictive value (PPV) of NIPT for SCAs remains variable, ranging from 32.4% to 86.7% (Deng et al., 2019; Hu et al., 2019; Zhang et al., 2017; La et al., 2021). Monosomy X has a relatively low PPV of 22%–29% (Martin et al., 2023; Petersen et al., 2017; Lüthgens et al., 2021), compared with other common sex chromosome trisomies (XXX, XXY, XYY). cfDNA analysis is based on fragmented DNA mainly derived from the placenta, it may not fully represent the complete fetal chromosomal constitution. Therefore, interpreting SCA-related NIPT results remains challenging, and discordant findings between cfDNA screening and the true fetal karyotype is able to occur. Also, Maternal chromosomal abnormalities, such as mosaicism and copy-number variations, may contribute to discordant NIPT results that actually reflect the underlying maternal karyotype rather than a true fetal abnormality (Grace et al., 2016; Dai et al., 2021).

Recently, the scope of NIPT has expanded to include clinically significant copy number variations (CNVs), such as microdeletions and microduplications. Representative syndromes detectable via expanded NIPT include DiGeorge syndrome (DGS), Prader–Willi/Angelman syndrome (PWS/AS), 22q11.22 microduplication, and cri-du-chat syndrome (CDC). Moreover, Several studies have evaluated the performance of NIPT in CNV detection (Li et al., 2024; Xue et al., 2022). Low-depth sequencing-based NIPT achieved a sensitivity of 81.58% for detecting CNVs >2 Mb, indicating its potential value as a screening tool (Ye et al., 2021). Despite ongoing evaluations, the clinical utility of NIPT in detecting SCAs and CNVs based on real-world data remains uncertain. In particular, the PPV of NIPT has been shown to vary with the type of SCA and CNV size (≤5 Mb, 5–10 Mb, >10 Mb) (Chen et al., 2019). Previous studies have reported a CNV detection rate of 51.1% and a PPV of 19.7% using NIPT (Wang et al., 2021). Therefore, a positive NIPT result for monosomy X and CNVs necessitates confirmatory invasive testing, such as fetal karyotyping and chromosomal microarray (CMA), more so than other aneuploidies. Together, these data suggest that beyond numerical SCAs, NIPT can indicate sex-chromosome structural abnormalities requiring confirmatory evaluation. We describe three such cases and their follow-up diagnostics to inform prenatal counseling.

## 2 Materials and methods

### 2.1 Study population and case identification

NIPT was routinely performed at our center between January 2022 and December 2024, during which a total of 10,025 pregnancies were screened. Among these, cases that screened positive for sex chromosome aneuploidies and subsequently underwent confirmatory invasive testing were reviewed. The three cases presented are those in which the final diagnostic work-up confirmed chromosomal structural abnormalities. This study was approved by the Institutional Review Board of CHA Gangnam Medical Center (IRB No. GCI 2025-07-006).

### 2.2 Sample preparation and NIPT

NIPT was performed using a massively parallel sequencing approach for whole-genome analysis. Maternal peripheral blood (10 mL) was collected in Cell-Free DNA BCT™ tubes (Streck, Omaha, NE, USA). Plasma was isolated by centrifugation at 1,200 × *g* for 10 min at 4 °C, followed by a second centrifugation at 16,000 × *g* for 10 min at 4 °C. Cell-free fetal DNA was extracted from 1 mL of plasma using the QIAamp Circulating Nucleic Acid Kit (Qiagen, Hilden, Germany). Library preparation was conducted using the Ion Plus Fragment Library Kit (Thermo Fisher, Waltham, CA, USA). Sequencing was performed on the Ion S5™ XL System (Life Technologies, Singapore) at an average depth of 0.3×. A total of 12–14 samples were processed per Ion 540™ Chip (Thermo Fisher Scientific). Each sample generated >5 million raw reads, with uniquely mapped reads exceeding 65.0%. The assay screened for common autosomal trisomies (T21, T18, T13), chromosomal microdeletions/duplications, and SCAs.

Z-scores for each chromosome were derived from normalized chromosomal representation. Whole-chromosome aneuploidy was determined using Z-score analysis, with |Z| ≥ 3 defined as high risk and −3 < Z < 3 considered low risk.

### 2.3 *FMR1* triplet-primed PCR (TP-PCR) and human androgen receptor (HUMARA) assay

To assess CGG repeat expansions in the *FMR1* gene (OMIM #309550), genomic DNA (gDNA) from amniocytes or peripheral blood was amplified using the LabGscan™ FRAXA PCR Kit (Labgenomics, Gyeonggi-do, Republic of Korea), following the manufacturer’s instructions. The HUMARA assay, a PCR-based method, was used to distinguish maternal and paternal X chromosome alleles and assess methylation status. As previously described (Allen et al., 1992), 0.5–1 μg of gDNA was digested with the methylation-sensitive restriction enzyme HpaII, followed by PCR amplification. PCR products were analyzed on the ABI 3500Dx Genetic Analyzer (Applied Biosystems, CA, USA) using GeneMapper software (Applied Biosystems).

### 2.4 Southern blot analysis targeting the *FMR1* locus

Southern blotting targeting the *FMR1* locus was employed to clarify ambiguous PCR results, such as homozygous patterns or failed amplification due to large repeat expansions. DNA (7–10 μg) was digested with EcoRI and EagI (New England Biolabs, Ipswich, MA, USA) (Gold et al., 2000). A biotin-labeled probe (StB12.3: forward 5′-CGC​CAA​GAG​GGC​TTC​AGG​TCT​CCT-3′; reverse 5′-GAG​ACT​GTT​AAG​AAC​ATA​AAC​GCG​GG-3′) was used for hybridization. Signal detection was performed using the Chemiluminescent Nucleic Acid Detection Module Kit (Thermo Fisher Scientific, Rockford, USA).

### 2.5 Quantitative fluorescent PCR (QF-PCR)

Fragment-based molecular assays were performed to assess clinically relevant copy-number changes and sex-chromosome abnormalities. Multiplex Ligation–Dependent Probe Amplification (MLPA) using SALSA probemixes P245 Microdeletion Syndromes-1A, P070 Subtelomeres Mix-2B, and P185 Intersex (MRC Holland, Amsterdam, Netherlands), following the manufacturer’s instructions. PCR products were analyzed using the ABI 3500Dx Genetic Analyzer and GeneMarker® software v3.0.1 (SoftGenetics, LLC, State College, PA, USA). Devyser Compact kit (Devyser, Hägersten, Sweden) for the rapid detection of trisomies 21, 18, and 13, and SCAs were performed according to the manufacturer’s protocols. Y-chromosome microdeletion testing targeted the azoospermia factor (AZF) region using standard sequence-tagged sites (STS) markers. Devyser Compact kit and Y-chromosome microdeletion testing amplified products were analyzed by capillary electrophoresis on the ABI 3500Dx platform and GeneMapper software v6 (Applied Biosystems).

### 2.6 Karyotyping and CMA

Karyotyping and CMA were performed on samples obtained via amniocentesis or chorionic villus sampling. Cells were cultured in BIO-AMF™ medium (Biological Industries, Cromwell, CT, USA), and metaphase spreads were prepared following standard G-banding protocols. A total of 20–30 metaphase cells were analyzed per sample. CMA was conducted using the Affymetrix CytoScan 750K array (Affymetrix, Santa Clara, CA, USA), comprising >750,000 oligonucleotide probes (including single-nucleotide polymorphism and non-polymorphic probes). Data were analyzed using the Chromosome Analysis Suite software (Affymetrix), based on the hg19 human genome assembly.

## 3 Case presentation

### 3.1 Case 1

**FIGURE 1:**
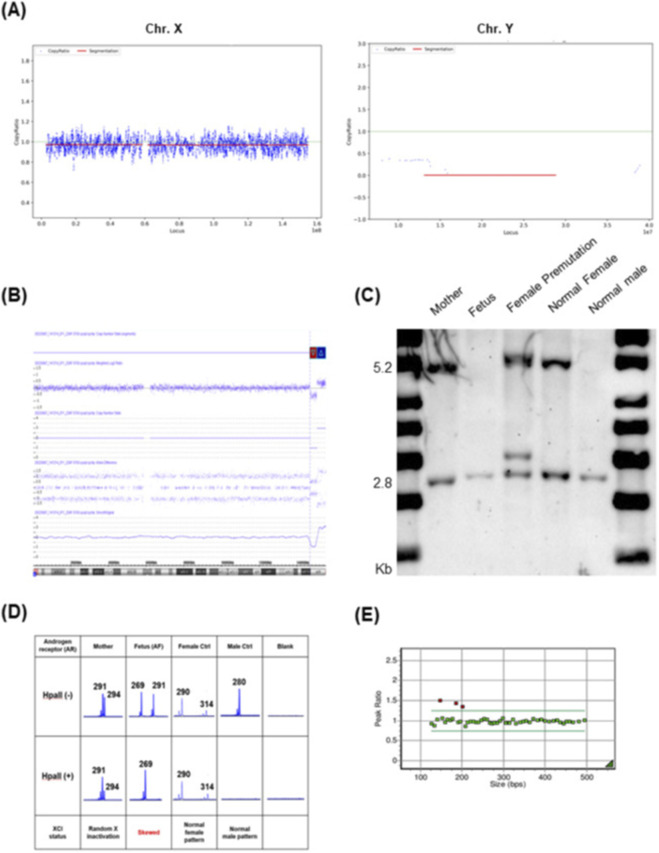
NIPT result suggestive of monosomy X-like pattern attributable to an X-chromosomal microdeletion. **(A)** NIPT result suggestive of monosomy X-like pattern. **(B)** Fetal Chromosomal microarray (CMA): 3.6 Mb deletion at Xq27.3q28 and 4.8 Mb duplication at Xq28. **(C)** Southern blot analysis; the mother (lane 1) showed a normal female pattern. However, the fetus (lane 2) showed only an active allele. **(D)** Human Androgen Receptor assay. **(E)** A duplication involving *MECP2* was detected by Multiplex Ligation-Dependent Probe Amplification (MLPA) using the P245 kit and is highlighted with a red box in the results.

A 38-year-old woman (G2P1) who conceived via *in vitro* fertilization (IVF) underwent NIPT at 12+1 weeks’ gestation due to advanced maternal age. Routine first-trimester ultrasonography revealed no fetal abnormalities. Maternal anthropometric characteristics, including weight, height, and BMI, were unremarkable. NIPT indicated a high risk for monosomy X (fetal fraction [FF] = 6.44%; X chromosome Z-score = −3.13) (Figure 1A). Following genetic counseling, karyotyping and CMA were performed at 16+3 weeks of gestation. The fetal karyotype was 46,XX, but CMA detected a 3.6 Mb deletion in the Xq27.3–q28 region [arr (Xq27.3q28) (146,806,191_150,386,543)×1] and a 4.8 Mb duplication in the Xq28 region [arr (Xq28) (150,408,165_155,233,731)×3] (Figure 1B). The maternal CMA result was normal.

To further clarify whether the *FMR1* region within this deleted segment was specifically affected, we performed *FMR1* TP-PCR and Southern blot analysis. TP-PCR fragment analysis indicated that both mother and fetus had homozygous normal alleles (29 CGG repeats). Southern blotting showed a normal female pattern in the mother (2.8 kb active and 5.2 kb inactive X chromosomes), while the fetus exhibited only the 2.8 kb band, indicating a single active X chromosome (Figure 1C). To assess whether the X chromosome harboring the *FMR1* deletion was subject to inactivation, the HUMARA assay was performed. Following digestion with the methylation-sensitive enzyme HpaII, only the paternally derived 269 bp allele was amplified in the fetus, indicating extremely skewed X-chromosome inactivation (XCI) (Figure 1D). The 4.8 Mb duplication in Xq28, encompassing *MECP2* (OMIM #300005), was confirmed by MLPA, providing concordant genomic support for the structural abnormality initially detected by CMA (Figure 1E). Paternal CMA was not performed. The Xq27.3–q28 deletion involved neurodevelopmentally relevant genes, including *FMR1* and *AFF2*, which have been associated with X-linked intellectual disability and cognitive/behavioral impairment (Wu and Li, 2022; Tekendo-Ngongang et al., 2021). In addition, the 4.8 Mb Xq28 duplication encompassed *MECP2*, overexpression of which is linked to *MECP2* duplication syndrome, characterized by severe neurodevelopmental deficits (Akaba and Takahashi, 2025). These potential impacts were discussed during genetic counseling, and the patient elected to terminate the pregnancy at 21 weeks of gestation.

### 3.2 Case 2

**FIGURE 2:**
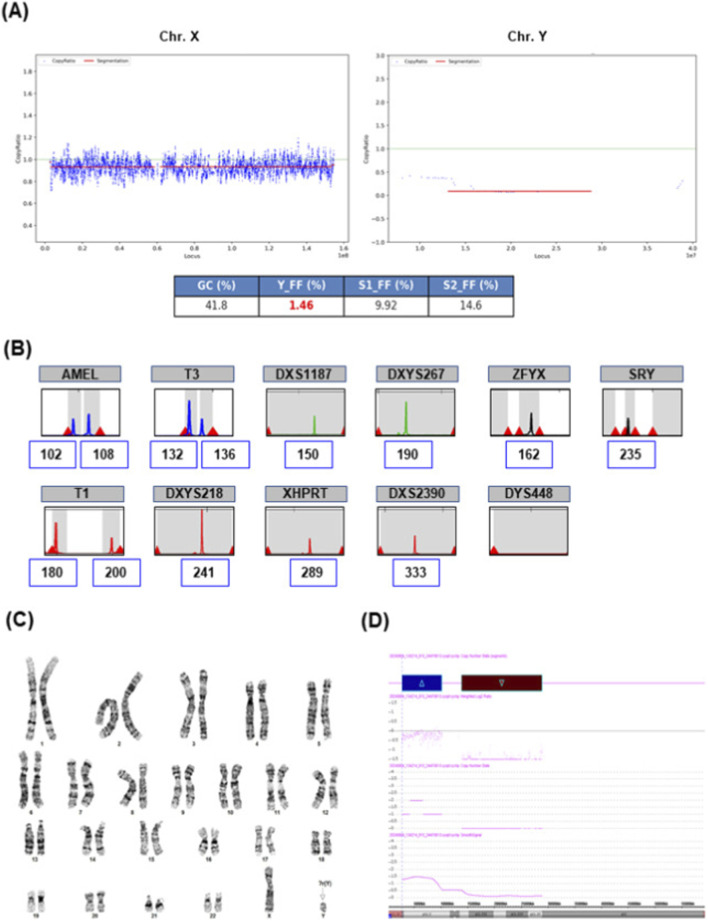
NIPT result suggestive of monosomy X with low Y signal attributable to mosaicism involving a ring Y chromosome. **(A)** NIPT result suggestive of monosomy X [fetal fraction = 9.92%, X chromosome Z-score = −8.54] and vanishing Twin pattern (Y_FF = 1.46, Y chromosome Z-score = 5.43). **(B)** Quantitative Fluorescence PCR (QF-PCR) results show short tandem repeats of sex chromosome. **(C)** Fetal karyotype; mos 46,X,?r(Y) (p11.32q11.223 [20]/45,X [9]. **(D)** The CMA result showed the duplication at Yp11.31p11.2 and deletion at Yq11.21q11.23.

A 33-year-old woman (G1P0) underwent NIPT at 12+0 weeks of gestation. NT measured 1.1 mm, with no ultrasonographic anomalies. NIPT indicated a high risk for monosomy X and a vanishing twin pattern (FF = 9.92% for the X chromosome, X chromosome Z-score = – 8.54; FF = 1.46% for the Y chromosome, Y chromosome Z-score = 5.43) (Figure 2A). At 16+0 weeks, QF-PCR, karyotyping, and CMA were performed. QF-PCR of amniocytes indicated an XY pattern, but the peak was absent at Y-specific markers ZFYX and DYS448 (Figure 2B). Karyotyping of cultured amniocytes revealed a mosaic karyotype: 46,X,?r(Y) (p11.32q11.223)[20]/45,X [9] (Figure 2C). CMA identified a 7.4 Mb duplication in Yp11.31–p11.2 region [arr (Yp11.31p11.2) (2,650,141_10,073,965)×1∼2] and a 15 Mb deletion in the Yq11.21–q11.23 region [arr (Yq11.21q11.23) (13,800,703_28,799,937)×0] (Figure 2D). Parental testing was not performed; thus, the origin of the ring chromosome remains unknown. Following genetic counseling, the patient opted for termination of pregnancy at 20+6 weeks.

### 3.3 Case 3

**FIGURE 3:**
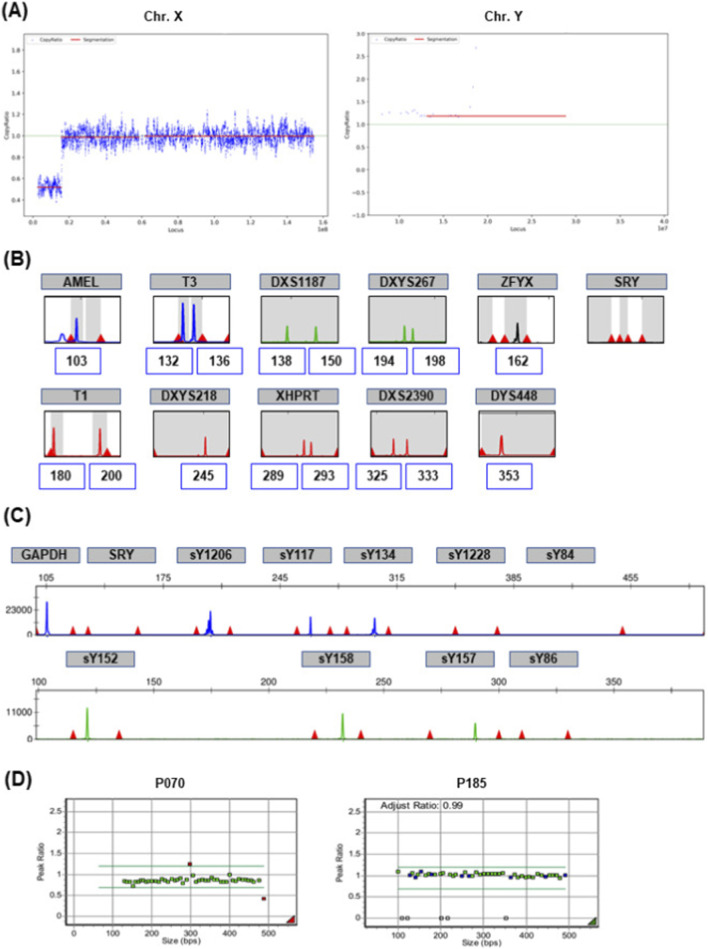
NIPT findings, interpreted considering molecular analysis results, attributable to an X–Y translocation in the maternal genotype. **(A)** NIPT detected a 12.97 Mb deletion in the ChrX (p22.33-p22.2) region. **(B)** Maternal QF-PCR showed signal detection at the DYS448 (Yq11.223). **(C)** Y chromosome microdeletion analysis. Ten sequence-tagged sites (STSs) were assessed: SRY, sY84, sY86, sY1228, sY117, sY134, sY152, sY157, sY158, and sY1206. **(D)** MLPA P070; single-copy loss of SHOX at pseudoautosomal region 1 (PAR1) and triplication of VAMP7 at PAR2. MLPA P185; Y chromosome-determining genes were indicated by a white box.

A 35-year-old woman (G1P0) underwent NIPT at 11+6 weeks of gestation. NT measured 1.1 mm, with no sonographic anomalies. The patient was underweight (height: 150 cm; weight: 41 kg; BMI: 18.2). Apart from being underweight, the mother did not present with any neurological, endocrine, gynecologic, or dysmorphic features of clinical concern. NIPT revealed a high risk for XYY (FF = 34.20%, X chromosome Z-score = – 2.61; Y chromosome Z-score = 6.46) and a 12.97 Mb microdeletion in the Xp22.33–p22.2 region (Figure 3A). After counseling, karyotyping, and CMA were performed on both the fetus and the mother at 16+5 weeks. QF-PCR of maternal blood showed a heterozygous pattern at the STR markers T3, DXS1187, T1, XHPRT, and DXS2390, indicating a normal female genotype (Figure 3B). However, the presence of a peak at the Y-specific DYS448 marker (Yq11.223) prompted Y chromosome microdeletion analysis to confirm the presence of the Y chromosome. The analysis identified six STSs in the AZFb region, each showing detectable peaks (sY117, sY134, sY152, sY157, sY158, and sY1206) (Figure 3C). MLPA revealed single-copy loss of *SHOX* (rsaXp22.33/Yp11.3 [SHOX]×1) and triplication of *VAMP7* (rsaXq28/Yq12 [VAMP7]×3) (Figure 3D).

**FIGURE 4:**
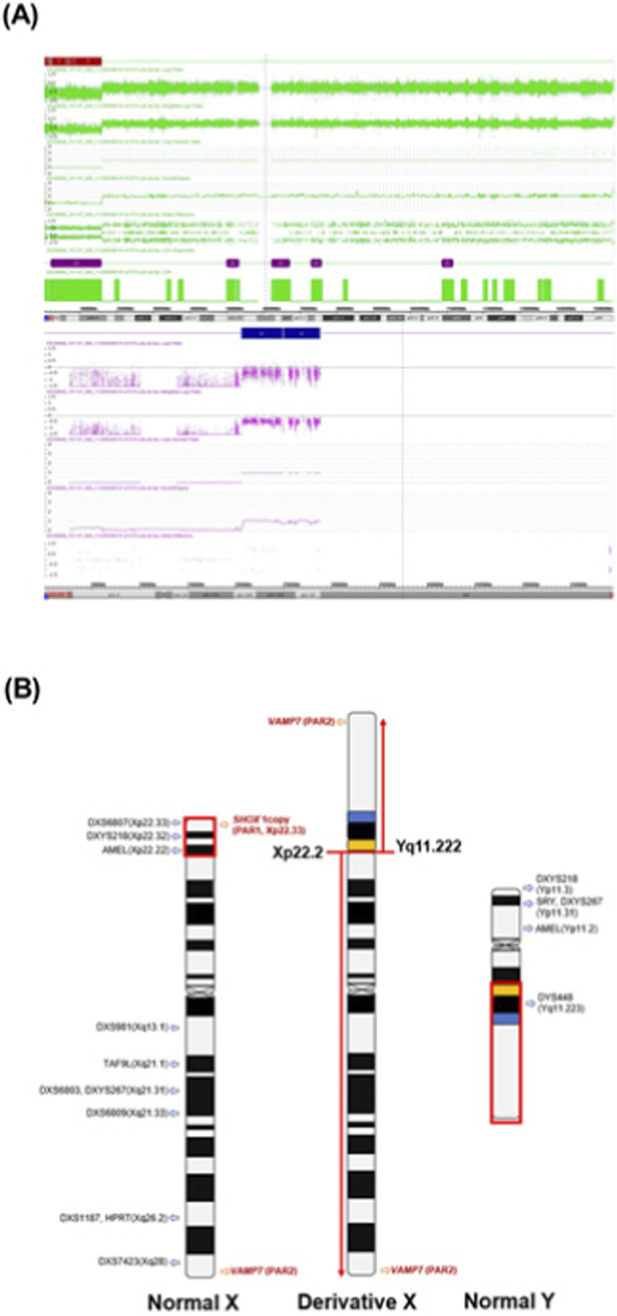
CMA findings and schematic representation of the inferred X–Y translocation. These data were derived from case 3. **(A)** Maternal CMA results show the deletion of X chromosome and the duplication of Y chromosome. Upper panel: Xp22.33p22.2 deletion, lower panel: Yq11.222q11.23 duplication. **(B)** Estimated maternal chromosome diagram of Xp; Yq unbalanced translocation. X and Y chromosome band nomenclature follows ISCN (2024).

Maternal karyotyping identified a translocation: 46,X,der(X)t (X; Y) (p22.31; q11.222). CMA revealed a 15.6 Mb deletion in the Xp22.33–p22.2 region [arr (Xp22.33p22.2) (168,546_15,766,553)×1] and an 8.2 Mb duplication in the Yq11.222–q11.23 region [arr (Yq11.222q11.23) (20,596,144_28,799,937)×1] (Figure 4A). These findings indicate that the estimated maternal chromosomal structure is 46,X,der(X)t (X; Y) (p22.2; q11.222) (Figure 4B). Invasive testing of the fetus confirmed a normal male genotype, and the pregnancy was continued.

Taken together, a concise summary of the NIPT results, invasive diagnostic findings, definitive diagnoses, and clinical relevance of the three cases is provided in Supplementary Table S1.

## 4 Discussion

NIPT can be utilized in the evaluation of a broad range of chromosomal abnormalities, including SCAs (Dowlut-McElroy et al., 2022). However, interpreting NIPT results for SCAs caused by structural abnormalities, such as deletions, ring chromosomes, and unbalanced translocations, requires caution due to factors like confined placental mosaicism (CPM) and maternal or fetal mosaicism (Dowlut-McElroy et al., 2022; Rudd et al., 2018; Xie et al., 2020). Especially, the presence of abnormal maternal chromosome variants can confound the test and contribute to false-positive results. In cohorts of discordant NIPT results for X-chromosome loss, maternal sex-chromosome mosaicism was identified in 36.7% of cases, suggesting that maternal mosaicism may contribute to some false-positive NIPT findings (Wan et al., 2022). In addition, CPM and maternal copy-number variants are well-recognized contributors to discrepancies between cfDNA screening results and the true fetal karyotype (Shaw et al., 2020).

In cases of monosomy X, the NIPT pattern may reflect either a true fetal XO or a maternal XO. Moreover, XO mosaicism or partial deletions of the X chromosome can produce a monosomy X–like result (Bedei et al., 2025). In particular, an Xq27.3 deletion may involve regulatory regions related to XCI (Marshall et al., 2013). As illustrated in our first case, deletions may involve critical regions such as XCI, depending on their location, and thus warrant careful interpretation.

In case 2, a specific pattern was observed, characterized by a low-level Y-chromosome fraction resembling the pattern typically seen in vanishing twin scenarios. Structural abnormalities of the Y chromosome have been reported to manifest on NIPT as a high-risk result for monosomy X, accompanied by a subtle increase in Y-chromosome representation (Xie et al., 2020). Similarly, a previous study has described cases where NIPT indicated a high risk for monosomy X due to the presence of a sex chromosome ring, with subsequent invasive testing confirming the presence of a ring X chromosome (Bedei et al., 2025). Y chromosomes are commonly associated with mosaicism due to segregation errors leading to chromosome loss or the formation of unstable ring structures (Henegariu et al., 1997). This instability may result in variable clinical phenotypes depending on the level of mosaicism and the extent of gene loss (Milenkovic et al., 2011).

NIPT results can be influenced by maternal genomic contribution, as the assay relies on cell-free DNA from both maternal and placental origins. In case 3, maternal 46,X,der(X)t (X; Y) (p22.2; q11.222) showed results on NIPT suggestive of XYY (Jacob’s syndrome), which likely reflected overrepresentation of X- and Y-chromosome fractions caused by the derivative-chromosome structure. A similar finding has been reported, involving an unbalanced X–Y translocation [der(X)t (X; Y) (p22.3; q11.2)] that was interpreted as XXY (Klinefelter syndrome) (Zhong et al., 2025). As a result, the same maternal der(X)t (X; Y) may appear on NIPT as either XXY or XYY, highlighting the importance of karyotyping to confirm the structural abnormality. Unbalanced X–Y translocations are often associated with terminal deletions of Xp, and the Xp22 region is recognized as a region prone to structural rearrangements, including deletions, duplications, and translocations (Daghsni et al., 2025). These translocations may have clinical implications when transmitted to the next-generation. In fact, several reports have described cases in which maternal der(X)t (X; Y) transmitted to the male fetus was associated with severe developmental delay and cutaneous abnormalities (Doherty et al., 2003; Metaxotou et al., 1983). These findings demonstrate that NIPT can indicate maternal structural abnormalities and highlight the need for genetic counseling tailored to fetal sex.

In addition to structural and placental aberrations, markedly abnormal or discordant cfDNA patterns may, in rare cases, reflect maternal malignancy rather than fetal aneuploidy. Although not observed in the case, this consideration suggests that NIPT results may incorporate both maternal and placental signals. Accordingly, unexplained findings warrant a cautious, multidisciplinary approach to interpretation (Moellgaard et al., 2022).

In summary, these three cases illustrate the challenges in interpreting NIPT results, highlighting the importance of considering sex chromosome structural abnormalities of either fetal or maternal origin in clinical interpretation. Nevertheless, the present work has certain limitations, including the small number of cases. Future studies in larger patient cohorts will be needed to further refine the evaluation of NIPT’s clinical utility and to establish evidence that can inform prenatal counseling and clinical decision-making.

## Data Availability

The original contributions presented in the study are included in the article/Supplementary Material, further inquiries can be directed to the corresponding authors.
