# Supplementary material for: Case Report: Expanding the diagnostic spectrum of non-invasive prenatal testing to structural chromosomal abnormalities
Source: Front Genet. 2026 Feb 17;17:1746287. doi: 10.3389/fgene.2026.1746287 (PMC12952721; doi:10.3389/fgene.2026.1746287)
Supplement: Supplementary file 1 [file Table1.pdf]

Supplementary Table S1. Overview of NIPT results, invasive diagnostic findings, final diagnoses, and clinical relevance of the three cases.

Case	NIPT Result	Invasive Diagnostic Findings	Final Diagnosis	Clinical / Counseling Relevance
Case 1	High-risk NIPT result for monosomy X	Amniocentesis; CMA identified deletion at Xq27.3–q28 and a duplication at Xq28;<br>HUMARA demonstrated skewed X-inactivation.	Xq27.3-q28 deletion and Xq28 duplication<br>with skewed X inactivation	Xq27.3–q28 deletion involving *FMR1* is associated with cognitive and behavioral neurodevelopmental impairment, whereas *MECP2* duplication at Xq28 is linked to a much more severe neurodevelopmental phenotype. These risks have important prognostic and counseling implications.
Case 2	High-risk NIPT result for monosomy X with a vanishing twin pattern	Karyotype: 46,X,?r(Y)/45,X mosaicism;<br>CMA: Yp duplication and Yq deletion consistent with ring Y formation.	Mosaic ring<br>Y chromosome	The clinical phenotype is highly variable, with potential risks including gonadal dysgenesis, impaired fertility, and endocrine dysfunction. Accordingly, endocrine evaluation and long-term clinical monitoring are recommended, particularly in male patients.
Case 3	High-risk NIPT result for XYY with a large Xp22.33-p22.2 microdeletion	Maternal testing: 46,X,der(X)t(X;Y);<br>fetal invasive testing normal	Maternal X-Y translocation	Sex-chromosome abnormalities on NIPT may reflect maternal structural rearrangements rather than fetal pathology.<br>Recognition of maternal origin is essential to avoid misinterpretation, provide accurate counseling, and assess recurrence risk.

Non-invasive prenatal testing (NIPT), Chromosomal microarray (CMA), Human androgen receptor assay (HUMARA)
